# Unipolar Polysaccharide-mediated Attachment of the N_2_O-reducing bacterium *Bradyrhizobium ottawaense* SG09 to Plant Roots

**DOI:** 10.1264/jsme2.ME25043

**Published:** 2025-11-14

**Authors:** Yudai Takeguchi, Ryota Shibuya, Momoi Kondo, Eriko Betsuyaku, Manabu Itakura, Kiwamu Minamisawa, Masayuki Sugawara, Shigeyuki Betsuyaku

**Affiliations:** 1 Graduate School of Agriculture, Ryukoku University, 1–5 Yokotani, Seta Oe-cho, Otsu, Shiga, 520–2194, Japan; 2 Faculty of Agriculture, Ryukoku University, 1–5 Yokotani, Seta Oe-cho, Otsu, Shiga, 520–2194, Japan; 3 Graduate School of Life Sciences, Tohoku University, 2–1–1 Katahira, Aoba-ku, Sendai, Miyagi, 980–8577, Japan; 4 Department of Life and Food Sciences, Obihiro University of Agriculture and Veterinary Medicine, West 2–11, Inada, Obihiro, Hokkaido 080–8555, Japan

**Keywords:** *Bradyrhizobium ottawaense*, N_2_O reduction, attachment, adhesin, unipolar polysaccharide

## Abstract

Agricultural soils are an important source of nitrous oxide (N_2_O), which has greenhouse and ozone-depleting effects. *Bradyrhizobium ottawaense* SG09 is a nitrogen-fixing rhizobium with high N_2_O-reducing activity. Rhizobia form symbiotic nodules in leguminous plants. The initial physical attachment of bacteria to plant roots is a critical step in the establishment of symbiotic interactions. In the present study, we performed a microscopic anal­ysis using DsRed-expressing *B. ottawaense* SG09. We revealed that *B. ottawaense* SG09 attached to both the root surface and root hairs via single cellular poles. This polar attachment was observed not only to the symbiotic host soybean, but also to non-leguminous plants, such as *Arabidopsis*, rice, corn, and wheat. We identified and analyzed the *unipolar polysaccharide* (*upp*) gene cluster, which is proposed to be involved in the polar attachment of rhizobia, in the genome of *B. ottawaense* SG09. We established an *Arabidopsis*-based interaction assay and demonstrated that *uppC* and *uppE* play a critical role in attachment to both the root surface and root hairs.

To establish beneficial symbiosis with legumes, rhizobia form symbiotic structures called nodules where they fix nitrogen. This nodulation process has been extensively exami­ned in *Medicago truncatula* and *Lotus japonicus*. Specifically, flavonoid compounds secreted by plant roots activate the rhizobial NodD transcriptional activator, which induces the production of Nod factors (Grundy *et al.*, 2023). When host plant cells perceive Nod factors, infection threads form in root hairs, rhizobia enter these threads, and nodulation occurs (Gage, 2004).

Prior to all these processes, the attachment of rhizobia to the host root surface is an important initial step. One of the most pronounced features of this process is the attachment of rhizobia to the cell surface of host roots via single cellular poles, as exemplified by previous studies on *Agrobacterium tumefaciens* (Matthysse, 2014). Bacteria utilize various adhesive molecules to attach to surfaces (Knights *et al.*, 2021). Among them, the polar adhesin unipolar polysaccharide (UPP), which is widely conserved in *Alphaproteobacteria*, is necessary for polar adhesion to both biotic and abiotic surfaces (Tomlinson and Fuqua, 2009; Li *et al.*, 2012; Fritts *et al.*, 2017). Components of the UPP biosynthesis pathway are separately encoded by the core *upp* gene cluster, called *uppABCDEF*, and the others in different regions of the genomes. A similar structure called holdfast, which is conserved in *Caulobacterales*, has been extensively exami­ned in *Caulobacter crescentus* (Berne *et al.*, 2015). The production of holdfast requires proteins encoded by the *hfs* gene cluster, called *hfsEFGHCBAD* (Smith *et al.*, 2003, Toh *et al.*, 2008). These two adhesins, UPP and holdfast, are distinct gene clusters independently conserved in the *Rhizobiales* and *Cladobacterium* clades, yet share some similarities (Fritts *et al.*, 2017). For example, *uppC* and *uppE* show high sequence similarity to *hfsD* and *hfsE*, respectively (Fritts *et al.*, 2017). Conservation of the *upp* gene cluster in plant-associated *Alphaproteobacteria*, such as *A. tumefaciens* and *Bradyrhizobium japonicum* suggests that UPP is important for plant-microbe interactions (Xu *et al.*, 2012, 2013; Fritts *et al.*, 2017). Previous studies demonstrated that UPP is required for the unipolar binding of rhizobia to plant lectins *in vitro*. The plant symbiont *Rhizobium leguminosarum* has been reported to utilize a UPP-type adhesin composed of glucomannan for attachment to pea and vetch roots, and this function is required for the competitive nodule infection of pea roots (Laus *et al.*, 2006; Williams *et al.*, 2008; Knights *et al.*, 2021).

*B. ottawaense* SG09 is a nitrogen-fixing rhizobacterium with high nitrous oxide (N_2_O)-reducing activity due to its high expression of the *nosZ* gene, which encodes N_2_O reductase (Wasai-Hara *et al.*, 2020, 2023). N_2_O is a long-lived greenhouse gas and ozone-depleting substance (Prather *et al.*, 2015). It is released from natural and anthropogenic sources, with more than half of the latter originating from agricultural soils (Tian *et al.*, 2020). The application of this bacterium is expected to effectively mitigate agricultural N_2_O emissions. However, attempts to infect legumes with specific rhizobial strains in agricultural fields often fail. This issue, which is due to competition between inoculants and indigenous rhizobia, highlights the importance of revealing the molecular basis of legume-rhizobia interactions (Thies *et al.*, 1991). In this context, the role of the aforementioned core *upp* gene cluster in *B. ottawaense* SG09 in the association with plants has never been experimental analyzed and, thus, remains unknown.

In the present study, we investigated the initial attachment of *B. ottawaense* SG09 to plant roots. We successfully visualized *B. ottawaense* SG09 using DsRed and revealed that this bacterium attached to the surface of root epidermal cells, including root hairs, via single cellular poles. This polar adhesion was observed not only in the interaction with the symbiotic host plant soybean, but also with non-leguminous plants, such as *Arabidopsis*, rice, corn, and wheat. We identified genes encoding the core *upp* gene cluster in the *B. ottawaense* SG09 genome and quantitatively evaluated their roles in the initial attachment to plant roots using deletion mutants of *uppC* and *uppE*.

## Materials and Methods

### Bacterial strains and growth conditions

*B. ottawaense* strains were grown aerobically at 30°C in HM salt medium (Cole and Elkan, 1973) supplemented with 0.1% arabinose and 0.025% (w/v) yeast extract. *Escherichia coli* strains were grown at 37°C in Luria–Bertani medium. The following antibiotics were added appropriately: 300‍ ‍mg L^–1^ neomycin (Nm) or 50‍ ‍mg‍ ‍L^–1^ polymyxin B (Px) for *B. ottawaense* SG09 and 50‍ ‍mg‍ ‍L^–1^ kanamycin for *E. coli*.

DsRed-expressing *B. ottawaense* SG09 (SG09-DsRed) was generated using the pUT-based mini-Tn5 vector pBjGroEL4::DsRed2 carrying the DsRed2 sequence under the control of the BjGroEL4 constitutive promoter (Hayashi *et al.*, 2014). All SG09-DsRed strains were grown in TY medium (5‍ ‍g‍ ‍L^–1^ tryptone, 3‍ ‍g‍ ‍L^–1^ yeast extract, and 1.3‍ ‍g‍ ‍L^–1^ CaCl_2_·2H_2_O) containing 100‍ ‍μg mL^–1^ spectinomycin at 28°C for 4–7 days.

### Construction of *uppE* and *uppC* deletion mutants

A mobilizable plasmid for generating the in-frame *uppE* deletion mutant (SG09DΔ*uppE*) was constructed as follows: a 0.9-kb fragment containing the 5′ flanking region of *uppE* (SG09_60840) and a fragment containing its 3′ flanking region were amplified by PCR from the genomic DNA of *B. ottawaense* SG09 using the primer sets uppE_mutF1/uppE_mutR1 and uppE_mutF2/uppE_mutR2 (Table S1), respectively. The two PCR products were fused by overlap extension PCR, and the fused fragment was cloned into the *Sma*I site of pK18*mobsacB* (Schäfer *et al.*, 1994) using an In-Fusion^®^ HD Cloning Kit (Takara Bio). The resulting plasmid (pMS195) was transferred from *E. coli* S17-1λpir to SG09-DsRed by biparental mating. An Nm/Px-resistant and sucrose-sensitive transconjugant was selected for single-crossover insertion of the plasmid into the chromosome. Cells were grown in HM liquid medium and spread on HM agar medium containing Px and 10% (w/v) sucrose to rescreen for sucrose-resistant colonies. The selected clones were further screened for Nm sensitivity. Double-crossover events and an in-frame deletion of *uppE* were confirmed by PCR using the primers uppE_F3/uppE_R3 (Table S1), and the nucleotide sequence of *uppE* in the mutants was elucidated by Eurofins Genomics based on the Sanger method.

To generate the in-frame *uppC* deletion mutant (SG09DΔ*uppC*), a mobilizable plasmid was constructed as follows: a 0.7-kb fragment containing the 5′ flanking region of *uppC* (SG09_08120) and a fragment containing its 3′ flanking region were amplified by PCR from the genomic DNA of *B. ottawaense* SG09 using the primer sets uppC_mutF1/uppC_mutR1 and uppC_mutF2/uppC_mutR2 (Table S1), respectively. The two PCR products were fused by overlap extension PCR, and the fused fragment was cloned into the *Sma*I site of pK18*mobsacB* as described above. The resulting plasmid (pMS196) was transferred from *E. coli* S17-1λpir to SG09-DsRed by biparental mating. The double-crossover mutants were selected as described above and confirmed by PCR using the primers uppC_F3/uppC_R3 (Table S1) and Sanger sequencing. The oligonucleotide primers used in this study are listed in Table S1.

### Plant growth conditions

Seeds of *Glycine max* cv. Enrei, *Arabidopsis thaliana* ecotype Col-0, *Oryza sativa* cv. Nipponbare, *Triticum aestivum* cv. Satonosora, and sweet corn (*Zea mays*) Wakuwaku Corn 82 Hybrid (Kaneko Seeds) were subjected to a microscopic anal­ysis. *Arabidopsis* seeds were surface-sterilized by soaking in 0.2% (v/v) Plant Preservative Mixture (Plant Cell Technology) at 4ºC for 1 day, and plated and grown on 1/2 MS media (M0404; Sigma-Aldrich) containing 0.3% phytagel (P8169; Sigma-Aldrich) at 23°C with 24‍ ‍h of light for the indicated number of days. The other seeds were sterilized by soaking them in approximately 1% sodium hypochlorite solution (Kitchen Haiter, Kao Corporation) followed by three rinses with an excess amount of sterile water and they were then grown on 1.5% agar plates at 25°C for the indicated number of days.

### Confocal microscopy of bacterial interactions with various plant roots

Three- to five-day-old seedings were inoculated. Bacterial cells grown on TY media were resuspended in sterile water and adjusted to an optical density at 600‍ ‍nm (OD_600_) of 0.003 for the inoculation of soybean (4-day-old), rice (4-day-old), *Arabidopsis* (5-day-old), and wheat (3-day-old) and an OD_600_ of 0.03 for the inoculation of sweet corn (3-day-old), unless otherwise specified (the overnight culture system for observing bacterial adhesion functions with bacterial suspensions having an OD_600_ in the range of 0.003 to 0.03.). The seedlings were incubated with 3‍ ‍mL of the bacterial suspension at the indicated concentrations in 35-mm cell culture dishes with tissue culture-treated surfaces (3000-035; IWAKI). The roots of overnight-treated seedlings were excised, washed once with sterile water, and mounted between the glass surface of a glass-bottomed dish (3910-035, IWAKI) and a 1/2 MS phytagel block. Fluorescence confocal and reflection imaging were performed using an LSM980 Airyscan 2 confocal microscope equipped with an LD LCI Plan-Apochromat 40×/1.2 Imm Corr DIC M27 objective and ZEN software (Carl Zeiss Microscopy).

### Quantification of bacterial attachment to the root surface

**Fig. 1.**
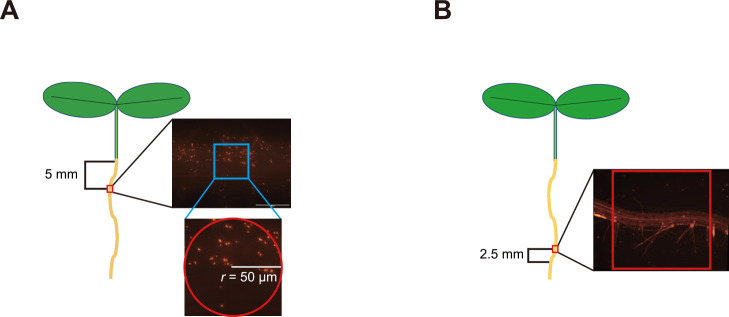
Schematic diagrams showing measurement methods used in this study. (A) Root surface attachment anal­ysis. A circle with a radius of 50‍ ‍μm centered on the root (shown in red) located 5‍ ‍mm from the first root hair in the microscopic image was used for bacterial counting. (B) Root hair attachment anal­ysis. The number of bacterial cells per root hair in a 1‍ ‍mm square centered on the root (shown in red) located 2.5‍ ‍mm from the first root hair from the root tip side was used for bacterial counting.

Five-day-old *Arabidopsis* ecotype Col-0 seedings were inoculated. Bacterial cells were prepared as described above. Six seedlings were submerged in 10‍ ‍mL of the bacterial suspension (OD_600_=0.003) in an Aznol Petri Dish JP, φ90×15‍ ‍mm (AS ONE) and incubated in this suspension at 23°C for 24‍ ‍h in the dark. Prior to the microscopic anal­ysis, seedlings were washed twice with sterile water. The root surface area located 5‍ ‍mm from the first root hair that appeared from the shoot side was selected for image acquisition using a BX53 fluorescence microscope equipped with a UPLFLN 40× objective and cellSens Standard software (Evident) (Fig. 1). A circle with a radius of 50‍ ‍μm centered on the root in the microscopic image was selected as the count area, while lateral roots and lateral root primordia were excluded, and it was exami­ned using Fiji (a distribution of ImageJ, version 2.14.0/1.54p) (Fig. 1A). Experiments were performed with six biological replicates and repeated three times individually.

### Quantification of bacterial attachment to root hairs

Four-day-old *Arabidopsis* ecotype Col-0 seedings were inoculated. Bacterial inoculation was performed as described above. After the inoculation, seedlings were incubated at 23°C for 3‍ ‍h in the dark. Prior to the microscopic anal­ysis, plants were washed twice with sterile water. An area of the root centered 2.5‍ ‍mm from the first root hair from the root tip side was selected for image acquisition using a BX53 fluorescence microscope equipped with a UPLFLN 10× objective and cellSens Standard software (Evident) (Fig. 1B). A square measuring 1×1‍ ‍mm^2^ centered on the root within the microscopic images was selected for counting using Fiji (a distribution of ImageJ, version 2.14.0/1.54g) (Fig. 1B). The number of bacteria per root hair was counted, and root hairs were classified into three groups based on the bacterial count: 0 (class I), 1–5 (class II), and 6 and more (class III). Experiments were performed with 3–6 biological replicates and repeated three times individually.

## Results

### *B. ottawaense* SG09 exhibits unipolar binding to the root surface

**Fig. 2.**
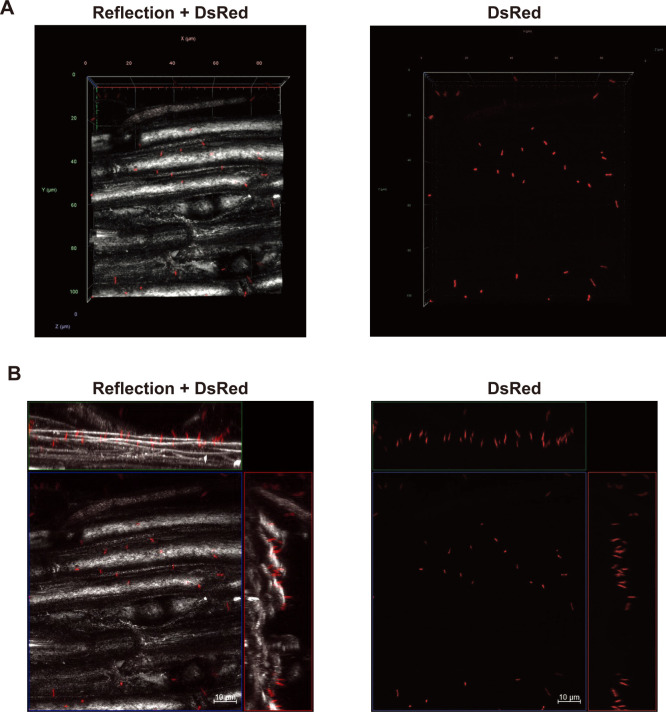
Confocal fluorescence imaging of roots of soybean seedlings incubated with SG09-DsRed. (A) Representative reconstituted 3D images of SG09-DsRed (red) binding to soybean roots visualized by CRM (gray). A merged image (left) and the corresponding DsRed image (right) are shown. (B) Orthogonal projection images of the reconstituted 3D image in (A) represented by the maximal projection. A merged image (left) and the corresponding DsRed image (right) are shown. Scale bar, 10‍ ‍μm.

**Fig. 3.**
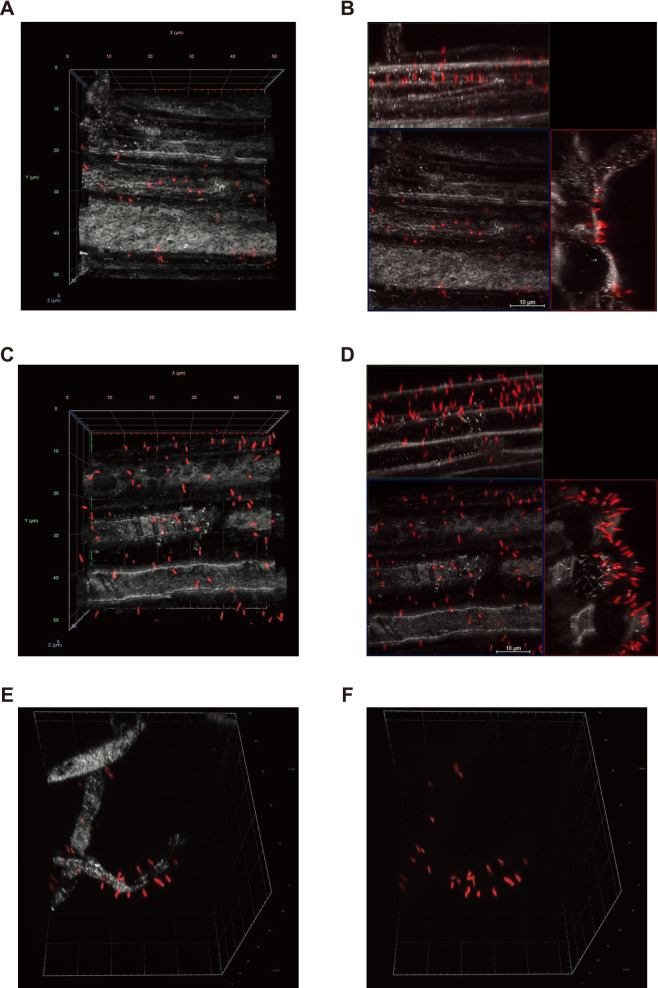
Confocal fluorescence imaging of roots of rice and *Arabidopsis* seedlings incubated with SG09-DsRed. (A) A representative reconstituted 3D image of SG09-DsRed (red) binding to rice roots visualized by CRM (gray). (B) An orthogonal projection image of the reconstituted 3D image in (A) represented by the maximal projection. (C) A representative reconstituted 3D image of SG09-DsRed (red) binding to *Arabidopsis* roots visualized by CRM (gray). (D) An orthogonal projection image of the reconstituted 3D image in (C) represented by the maximal projection. (E) Representative reconstituted 3D images of SG09-DsRed (red) binding to rice root hairs visualized by CRM (gray). (F) A DsRed image of the 3D images shown in (E). Scale bars, 10‍ ‍μm.

To gain insights into the initial physical interaction between *B. ottawaense* SG09, which has high N_2_O-reducing activity, and plant root tissues, we focused on soybean, a potential agricultural target for the practical application of this bacterium. Rhizobial attachment to legume roots is affected by environmental factors, such as soil pH (Knights *et al.*, 2021). To simplify the comparison, we developed a simple incubation assay using aseptically germinating soybean seedlings together with *B. ottawaense* SG09 carrying chromosomally integrated DsRed2 under the control of a constitutive promoter (SG09-DsRed) in sterile water. Confocal laser scanning microscopy (CLSM) and confocal reflection microscopy (CRM) were employed to visualize DsRed fluorescence from SG09-DsRed cells and non-fluorescently labeled plant tissue structures, respectively, allowing reconstruction of the three-dimensional (3D) structure of bacterial attachment to plant tissues (Paddock, 2002; Kiyokawa *et al.*, 2017). After an incubation for 24 h, the roots were washed and analyzed. CLSM and CRM revealed that multiple SG09-DsRed cells adhered to the soybean root epidermal surface via single poles, resembling the well-known characteristics of rhizobia (Fig. 2A and Supplementary Movie 1) (Fritts *et al.*, 2017). An orthogonal projection of the reconstituted 3D structure clearly visualized the unipolar attachment of individual SG09-DsRed cells to the plant cell surface (Fig. 2B). We then investigated whether this unipolar adhesion to the plant cellular surface was specific to soybean, which *B. ottawaense* SG09 establishes a symbiotic relationship with through nodulation (Wasai-Hara *et al.*, 2020). When similarly incubated with the roots of rice, wheat, sweet corn, and even *Arabidopsis*, SG09-DsRed cells attached to all the roots exami­ned via single cellular poles (Fig. 3 and S1, Supplementary Movies 2 and 3). This unipolar attachment was not only observed to root epidermal surfaces, but also to root hairs (an example of rice root hairs is shown in Fig. 3D and E, Supplementary Movie 4). These results show that *B. ottawaense* SG09 adhered to the cellular surfaces of various plant species without any specific preference.

### The core *upp* gene cluster is conserved in the *B. ottawaense* SG09 genome

UPP was previously reported to be required for bacterial polar attachment; therefore, we investigated whether *B. ottawaense* SG09 possesses homologous genes to *uppABCDEF* of *Rhodopseudomonas palustris* CGA009 (Fritts *et al.*, 2017; Onyeziri *et al.*, 2022). A BLASTP anal­ysis identified homologous genes in the *B. ottawaense* SG09 genome for each of the *R. palustris* CGA009 genes, and these homologs were consequently designated *uppA*, *uppB*, *uppC*, *uppD*, *uppE*, and *uppF* in *B. ottawaense* SG09 (Fig. S2A, Table S2). The core *upp* gene cluster in the *B. ottawaense* SG09 genome consists of *uppABDEF*, while *uppC* is located separately, showing synteny with that of *B. japonicum* USDA 110 (Fritts *et al.*, 2017). *uppC* and *uppE* in *R. palustris* and *A. tumefaciens* play a crucial role in the production of UPP; therefore, we generated SG09-DsRed in-frame deletion mutants of *uppC* and *uppE*, called SG09DΔ*uppC* and SG09DΔ*uppE* (Fig. S2B and C) (Fritts *et al.*, 2017; Onyeziri *et al.*, 2022).

### *uppC* and *uppE* are required for the attachment of *B. ottawaense* SG09 to plant surfaces

**Fig. 4.**
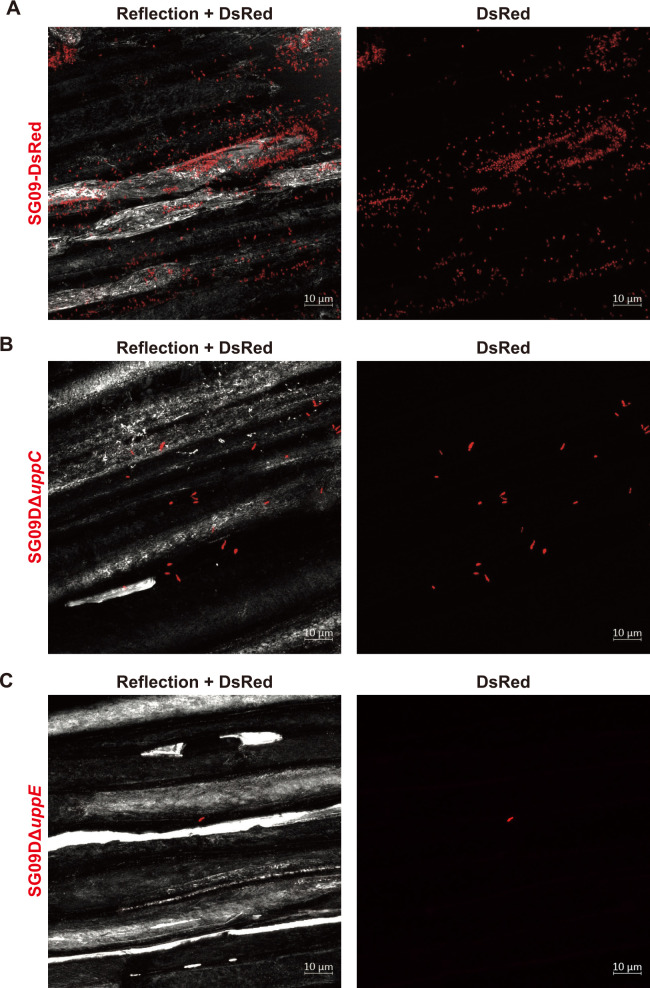
Confocal fluorescence imaging of soybean roots incubated with SG09-DsRed, SG09DΔ*uppC*, and SG09DΔ*uppE*. Soybean seedlings were incubated with a suspension of the designated bacterial strain (OD_600_=0.02) of the indicated bacterial strains for 24 h, washed, and then observed using CLSM and CRM. (A) Representative maximum intensity projection (*xy* plane) of SG09-DsRed (red) binding to a soybean root visualized by CRM (gray). A merged image (left) and the corresponding DsRed image (right) are shown. (B) Representative maximum intensity projection (*xy* plane) of SG09DΔ*uppC* (red) binding to a soybean root visualized by CRM (gray). A merged image (left) and the corresponding DsRed image (right) are shown. (C) Representative maximum intensity projection (*xy* plane) of SG09DΔ*uppE* (red) binding to a soybean root visualized by CRM (gray). A merged image (left) and the corresponding DsRed image (right) are shown. Scale bars, 10‍ ‍μm.

**Fig. 5.**
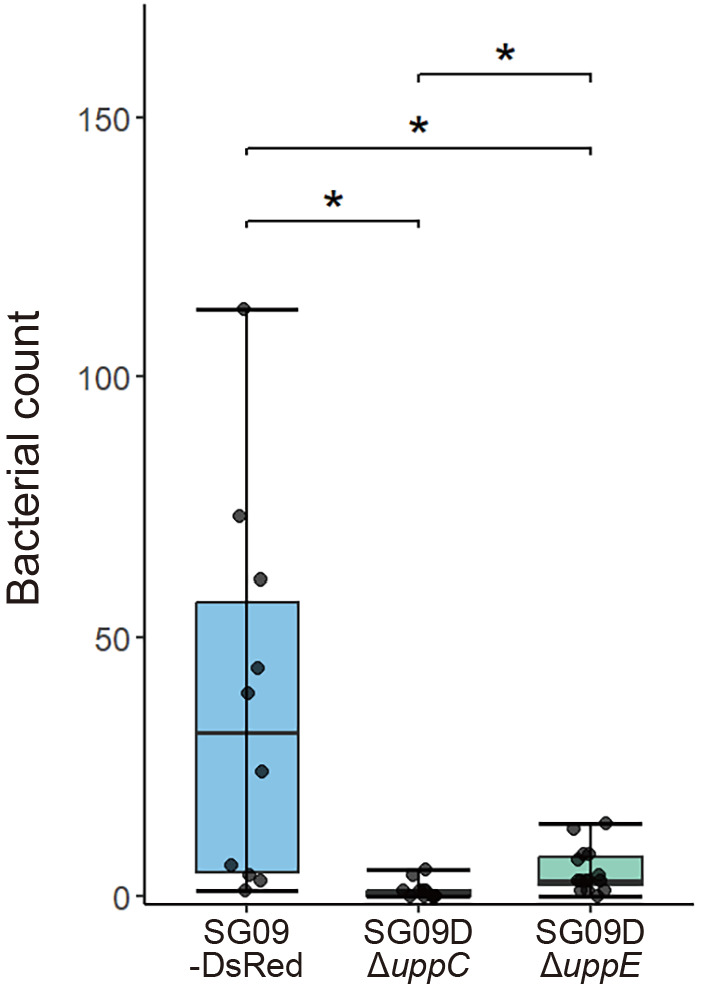
Quantification of bacterial attachment to the root surface. Five-day-old Col-0 seedlings were incubated with bacterial suspensions (OD_600_=0.003). After an incubation at 23°C for 24‍ ‍h in the dark, plants were washed twice with sterile water. The number of epidermally attached bacterial cells present within a circle with a radius of 50‍ ‍μm set 5‍ ‍mm away from the first root hair observed from the shoot side was counted. *n*=10 for SG09-DsRed, *n*=13 for SG09DΔ*uppC*, and *n*=15 for SG09DΔ*uppE*. *P<0.05. Statistical anal­yses were performed by the *t*-test using R (version 4.4.2).

A functional anal­ysis of bacterial mutants in terms of their polar attachment is often performed using *in vitro* assays, such as biofilm formation and lectin-binding assays. The use of cultured soybean cells and a specific antibody against a *B. japonicum* strain enabled a more direct and quantitative binding assay with plant cells; however, it is costly and labor-intensive to raise specific antibodies and maintain cell cultures (Ho *et al.*, 1988). We initially investigated the effects of both mutations using our simple incubation assay employing CLSM and CRM. After incubating soybean roots with these bacterial strains for 24 h, we revealed that both mutations resulted in fewer attachments to the roots than the wild type (Fig. 4). However, it was extremely difficult to quantitatively evaluate the adhesion rate of *upp* mutants that show little to no adhesion, but occasionally adhere to soybean roots, which are markedly larger than the size of the bacteria, without bias within the field of view where bacteria may be identified under a microscope. Therefore, since SG09-DsRed exhibits unipolar attachment to the roots of the small plant, *Arabidopsis*, in addition to soybean, we microscopically investigated the roles of *uppC* and *uppE* using *Arabidopsis* ecotype Col-0 as a host in a small-scale, direct, and quantitative attachment assay. The use of *Arabidopsis* seedling roots enabled the coverage of nearly the entire root width at specific locations, allowing for a quantitative anal­ysis with minimal positional bias. We designed two assays to characterize SG09DΔ*uppC* and SG09DΔ*uppE*, along with SG09-DsRed, in terms of epidermal and root hair attachment (see Materials and Methods). Although the number of SG09-DsRed that attached to the root surface markedly varied among root samples, significantly fewer SG09DΔ*uppC* and SG09DΔ*uppE* than SG09-DsRed attached (Fig. 5). Additionally, significantly fewer SG09DΔ*uppC* than SG09DΔ*uppE* attached to the root surface (Fig. 5).

**Fig. 6.**
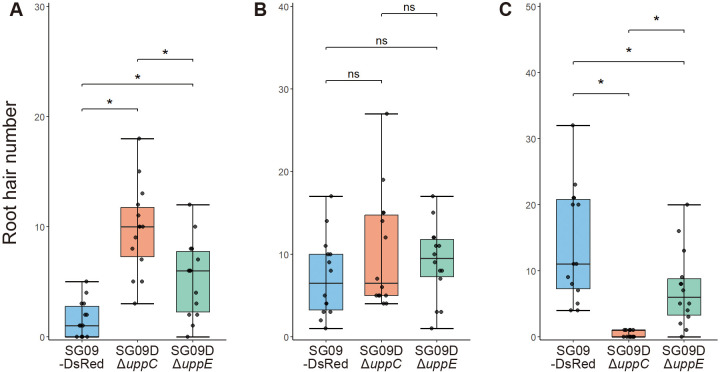
Quantification of bacterial attachment to root hairs. Four-day-old Col-0 seedlings were incubated with bacterial suspensions (OD_600_=0.3). After an incubation at 23°C for 3‍ ‍h in the dark, plants were washed twice with sterile water. A 1‍ ‍mm square centered 2.5‍ ‍mm from the first root hair from the root tip side was used for bacterial counting. The number of bacterial cells attached to each root hair was counted and root hairs were then classified into three groups based on the number of attached bacteria: 0 (A, class I), 1–5 (B, class II), and 6 and more (C, class III). *n*=14. *P<0.05; ns, not significant. Statistical anal­yses were performed by the *t*-test using R (version 4.4.2).

We then focused on root hair attachment. The number of bacteria that attached to root hairs markedly varied; therefore, root hair attachment was analyzed by classifying the observed phenotypes into three categories based on the number of attached bacterial cells: 0 (class I), 1–5 (class II), and 6 and more (class III). The number of class I root hairs was significantly higher (Fig. 6A), while the number of class III root hair was significantly lower (Fig. 6C) in both mutants than in SG09-DsRed. In terms of the numbers of class I and class III root hairs, SG09DΔ*uppC* had a significantly more severe phenotype than SG09DΔ*uppE* (Fig. 6A and C). The number of class II root hairs did not significantly differ among the tested strains, suggesting that this class reflected the accidental attachment of bacterial cells to root hairs (Fig. 6B). The results of our quantitative microscopic anal­ysis revealed that UppC and UppE play a direct and essential role in the attachment of *B. ottawaense* SG09 cells to the root epidermal surface and root hairs.

## Discussion

We herein demonstrated that *B. ottawaense* SG09 attached to both the root surface and root hairs via single cellular poles. This attachment was observed not only to soybean, a symbiotic leguminous host for this bacterium, but also to non-leguminous host plants, such as *Arabidopsis*, rice, corn, and wheat. We showed that the *upp* gene cluster was conserved in the *B. ottawaense* SG09 genome. The disruption of *uppC* and *uppE* in *B. ottawaense* SG09 reduced the attachment of the bacterium to the roots of soybean and *Arabidopsis*. The difficulties associated with performing microscopic and unbiased quantitative anal­yses of physical direct interactions between soybean roots and bacteria, which markedly differ in size, were overcome by establishing a small-scale assay using *Arabidopsis*, which revealed a significant reduction in the attachment of this bacterium to both the root surface and root hairs in *Arabidopsis*. Our assay using *Arabidopsis* provides a high-throughput approach *in planta*. In addition, it enables us to use several genetic tools, *e.g.*, various well-known mutants of plant lectins and immune system components, to molecularly elucidate the interactions of *B. ottawaense* SG09 with plants.

There is a marked phenotypic difference between SG09DΔ*uppC* and SG09DΔ*uppE*, suggesting that UppC plays a crucial role in attachment to both the root surface and root hairs. *uppC* and *uppE* encode the Wza-type outer membrane polysaccharide secretin and WbaP-initiating glycosyltransferase, respectively, in *A. tumefaciens* (Onyeziri *et al.*, 2022). In *A. tumefaciens*, *uppE* is necessary for UPP production under inorganic phosphate-rich conditions, while *uppE* and its paralog *Atu0102* act redundantly under inorganic phosphate-limited conditions (Xu *et al.*, 2012). Therefore, other genes may function redundantly with *uppE* in the *B. ottawaense* SG09 genome. The present results suggest that UPP is important for the attachment of *B. ottawaense* SG09 to biotic surfaces. Further information is essential for elucidating the function of UPP in *B. ottawaense* SG09, *e.g.*, the biochemical composition that governs lectin specificity (Onyeziri *et al.*, 2022). However, it is difficult to elucidate the biochemical composition of UPP because of its insolubility and low level of synthesis by bacterial cells (Berne *et al.*, 2015; Thompson *et al.*, 2018).

*B. ottawaense* SG09 is gaining attention because of its high N_2_O-reducing activity (Wasai-Hara *et al.*, 2023). Further optimization is required to exploit *B. ottawaense* SG09 as an N_2_O-reducing agent in agricultural fields, including overcoming the competitive effects of indigenous bacteria (Thies *et al.*, 1991; Wasai-Hara *et al.*, 2023). The initial process of bacterial attachment to the root surface may be an important target for inoculated bacteria to successfully dominate during nodulation. In the present study, we demonstrated the involvement of UPP in the initial attachment of *B. ottawaense* SG09 to the roots of various plant species and established a small-scale, direct, and quantitative *in planta* assay using *Arabidopsis* as a host, which will broaden our knowledge of the mechanisms underlying rhizobial attachment. Further research may lead to the engineering of rhizobial adhesins that improve the probability of attachment and the development of methods that overcome indigenous bacteria.

## Citation

Takeguchi, Y., Shibuya, R., Kondo, M., Betsuyaku, E., Itakura, M., Minamisawa, K., et al. (2025) Unipolar Polysaccharide-mediated Attachment of the N_2_O-reducing bacterium *Bradyrhizobium ottawaense* SG09 to Plant Roots. *Microbes Environ ***40**: ME25043.

https://doi.org/10.1264/jsme2.ME25043

## Supplementary Material

Supplementary Material 1

Supplementary Material 2

Supplementary Material 3

Supplementary Material 4

Supplementary Material 5
