# Supplementary material for: Unipolar Polysaccharide-mediated Attachment of the N2O-reducing bacterium Bradyrhizobium ottawaense SG09 to Plant Roots
Source: Microbes Environ. 2025 Nov 14;40(4):ME25043. doi: 10.1264/jsme2.ME25043 (PMC12727192; doi:10.1264/jsme2.ME25043)
Supplement: Supplementary file 1 — Supplementary Material 1 [file 40_25043_s1.pdf]

Supplementary Materials for:

Unipolar polysaccharide-mediated attachment of the N_2O-reducing bacterium *Bradyrhizobium ottawaense* SG09 to plant roots.

Authors:

Yudai Takeguchi[1], Ryota Shibuya[2], Momoi Kondo[2], Eriko Betsuyaku[2], Manabu Itakura[3], Kiwamu Minamisawa[3], Masayuki Sugawara[4*], and Shigeyuki Betsuyaku[2*]

Figure S1.

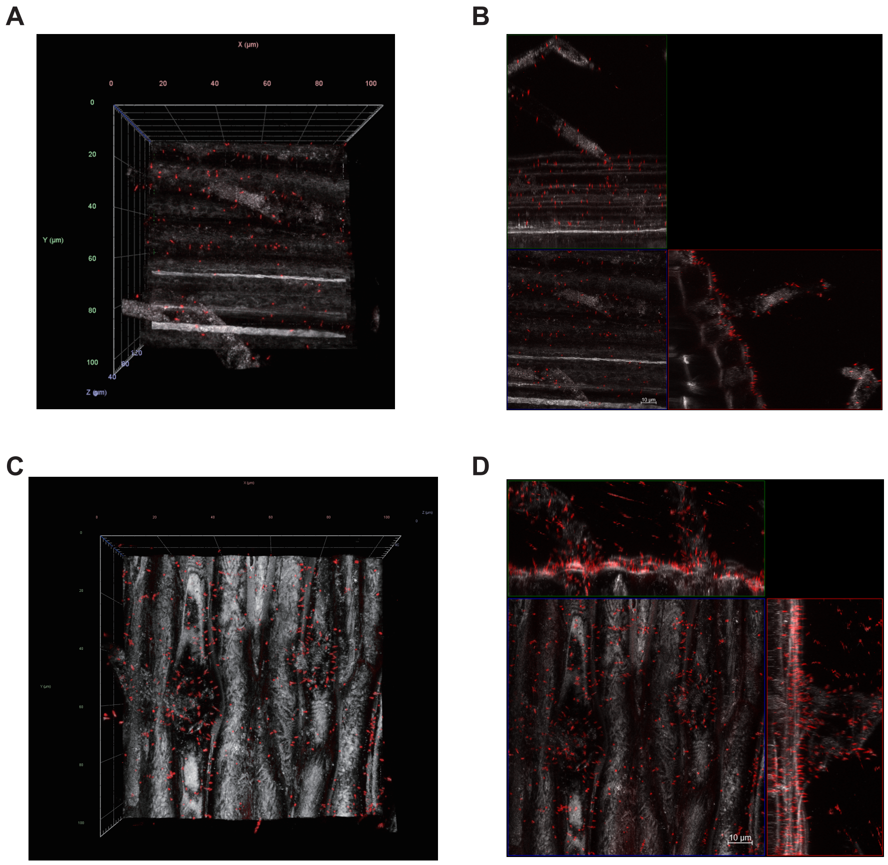

Figure S1. Confocal fluorescence imaging of the roots of wheat and sweet corn seedlings incubated with SG09-DsRed.

(A) A representative reconstituted 3D image of SG09-DsRed (red) binding to wheat roots visualized by CRM (gray). (B) An orthogonal projection image of the reconstituted 3D image in (A) represented by maximal projection. (C) A representative reconstituted 3D image of SG09-DsRed (red) binding to sweet corn roots visualized by CRM (gray). (D) An orthogonal projection image of the reconstituted 3D image in (C) represented by maximal projection. Scale bars, 10 μm.

Figure S2. Takeguchi et al

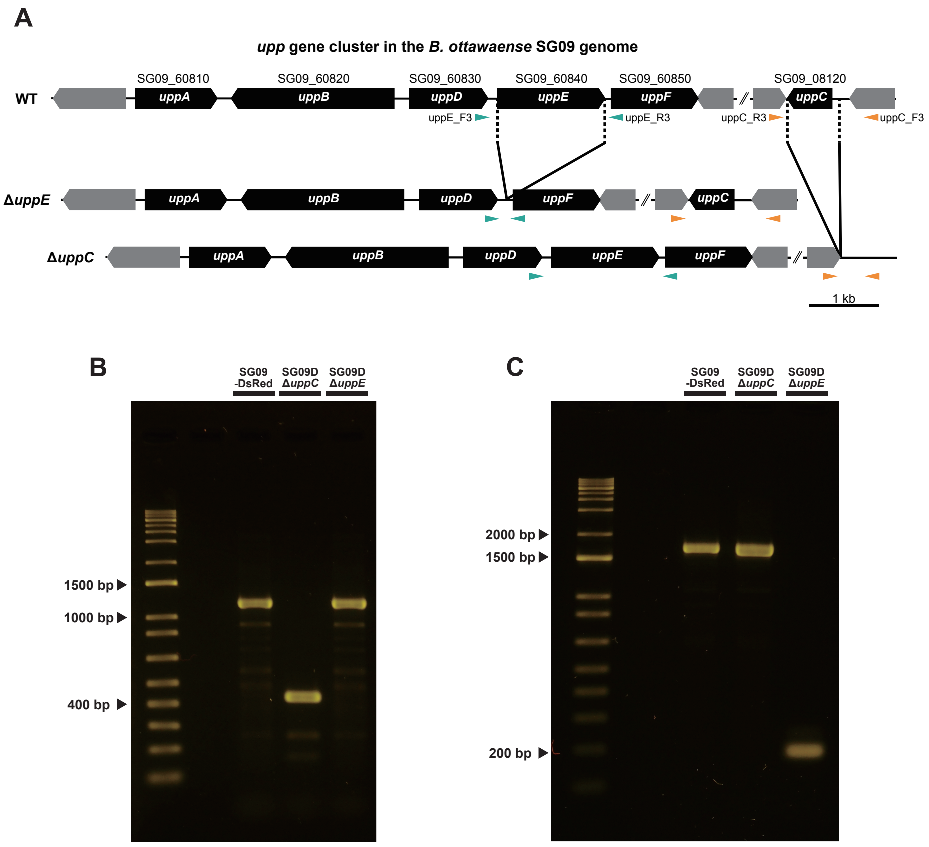

Figure S2. The core *upp* gene cluster in the *B. ottawaense* SG09 genome and the mutants generated in this study.

(A) Schematic representations of the *upp* gene cluster in the genomes of wild-type *B. ottawaense* SG09 and the respective mutants. (B, C) PCR validation of SG09DΔ*uppC* and SG09DΔ*uppE* loci using the primer pairs uppC_F3/uppC_R3 for *uppC* (B) and uppE_F3/uppE_R3 for *uppE* (C). PCR products were separated on 2% agarose gels.

Supplementary Movie 1. Reconstituted 3D rendering of SG09-DsRed (red) binding to soybean roots visualized by CRM (gray) shown in Fig. 1A.

Supplementary Movie 2. Reconstituted 3D rendering of SG09-DsRed (red) binding to rice roots visualized by CRM (gray) shown in Fig. 2A.

Supplementary Movie 3. Reconstituted 3D rendering of SG09-DsRed (red) binding to *Arabidopsis* roots visualized by CRM (gray) shown in Fig. 2C.

Supplementary Movie 4. Reconstituted 3D rendering of SG09-DsRed (red) binding to rice root hairs visualized by CRM (gray).

Table S1.

Table S1. Primers used in this study

Primer name	Sequence (5' to 3')
uppC_mutF1	TCGAGCTCGGTACCCCGACCACCCATTTCGTCAAC
uppC_mutR1	TGGATTTCAGGCGGTGAGCGAGACGGCTTAAGTGATGC
uppC_mutF2	GCATCACTTAAGCCGTCTCGCTCACCGCCTGAAATCCA
uppC_mutR2	CTCTAGAGGATCCCCTTTGAAATAGGGCGCGTCTC
uppC_F3	CCTATTCCGATGCGCAGAAG
uppC_R3	CATCGACGCTAACAGGATCG
uppE_mutF1	TCGAGCTCGGTACCCCGCTGCATTATCCCCTCAAC
uppE_mutR1	CGGGGCATGACGGAAAACTCCACGTCCATTTCAAGCAC
uppE_mutF2	GTGCTTGAAATGGACGTGGAGTTTTCCGTCATGCCCCG
uppE_mutR2	CTCTAGAGGATCCCCGTGGAATCGAACGACAGCAG
uppE_F3	TAACCATCCTTAAGCCCGCT
uppE_R3	CTATTCGGACGCCAAACACC

*The underlined sequences are homologous regions of pK18mobsacB.

Table S2.

Table S2. BLASTP analysis of the orthologous genes of *uppABCDEF* in *B. ottawaense* SG09

***Rhodopseudomonas palustris* CGA009**	***Bradyrhizobium ottawaense* SG09**		
	**Locus tag**	**Position**	**Identity(%)**
RPA2753_UppA	SG09_60810	6502658-6503839	230/390 (59%)
RPA2752_UppB	SG09_60820	6504021-6506357	484/699 (69%)
RPA2751_UppD	SG09_60830	6506563-6507696	262/377 (69%)
RPA2750_UppE	SG09_60840	6507817-6509364	401/519 (77%)
RPA4581_UppF	SG09_60850	6509436-6510692	227/404 (56%)
RPA4833_UppC	SG09_08120	888458-889111	143/217 (66%)
